# Epigenetic activation of *MGAT3* and corresponding bisecting GlcNAc shortens the survival of cancer patients

**DOI:** 10.18632/oncotarget.10543

**Published:** 2016-07-12

**Authors:** Reto S. Kohler, Merrina Anugraham, Mónica Núñez López, Christina Xiao, Andreas Schoetzau, Timm Hettich, Goetz Schlotterbeck, André Fedier, Francis Jacob, Viola Heinzelmann-Schwarz

**Affiliations:** ^1^ Ovarian Cancer Research, Department of Biomedicine, University Hospital Basel and University of Basel, Basel, Switzerland; ^2^ School of Life Sciences, University of Applied Sciences and Arts Northwestern Switzerland, Muttenz, Switzerland; ^3^ Glyco-Oncology, Ovarian Cancer Research, Department of Biomedicine, University Hospital Basel and University of Basel, Basel, Switzerland; ^4^ Hospital for Women, Department of Gynecology and Gynecological Oncology, University Hospital Basel, Basel, Switzerland

**Keywords:** N-glycosylation, DNA methylation, long-time survival, bisecting GlcNAc, ovarian cancer

## Abstract

Bisecting GlcNAc on *N*-glycoproteins is described in E-cadherin-, EGF-, Wnt- and integrin- cancer-associated signaling pathways. However, the mechanisms regulating bisecting GlcNAc expression are not clear. Bisecting GlcNAc is attached to *N*-glycans through beta 1-4 *N*-acetylglucosaminyl transferase III (MGAT3), which is encoded by two exons flanked by high-density CpG islands. Despite a recently described correlation of *MGAT3* and bisecting GlcNAc in ovarian cancer cells, it remains unknown whether DNA methylation is causative for the presence of bisecting GlcNAc. Here, we narrow down the regulatory genomic region and show that reconstitution of *MGAT3* expression with 5-Aza coincides with reduced DNA methylation at the *MGAT3* transcription start site. The presence of bisecting GlcNAc on released *N*-glycans was detected by mass spectrometry (LC-ESI-qTOF-MS/MS) in serous ovarian cancer cells upon DNA methyltransferase inhibition. The regulatory impact of DNA methylation on *MGAT3* was further evaluated in 18 TCGA cancer types (*n* = 6118 samples) and the results indicate an improved overall survival in patients with reduced MGAT3 expression, thereby identifying long-term survivors of high-grade serous ovarian cancers (HGSOC). Epigenetic activation of *MGAT3* was also confirmed in basal-like breast cancers sharing similar molecular and genetic features with HGSOC. These results provide novel insights into the epigenetic regulation of *MGAT3*/bisecting GlcNAc and demonstrate the importance of *N*-glycosylation in cancer progression.

## INTRODUCTION

High-grade serous ovarian cancer (HGSOC) is the most lethal gynecological cancer in women and despite improved surgical techniques and drug regimens, overall survival has not changed significantly for several decades [1]. Intriguingly, there are individuals who live on for more than 10 years after diagnosis, also known as long-term survivors. However, the molecular and cellular mechanisms underlying this long-term survivorship are still unknown despite the presence of more long-term survivors than previously expected [2]. Hence, the identification of molecular pathways of long-term survival could significantly advance treatment options of HGSOC.

Almost all cancer biomarkers used in the clinics nowadays are secreted glycoproteins [3]. In particular, in ovarian cancer CA125 is the most commonly used tumor marker and is glycosylated at threonine (T) or serine (S) and asparagine (N), resulting in *O*- and *N*-glycopeptides, respectively [4]. The synthesis of *N*-glycosylation at the consensus sequence, Asn-X-Ser/Thr, of a polypeptide chain involves a complex biosynthetic pathway in which the core glycan Glc_3_Man_9_GlcNAc_2_ is trimmed by several glycosidases to form high mannose *N*-glycans, and further processed in the Golgi apparatus by the sequential action of GlcNAc T-I (MGAT1) to form hybrid *N*-glycans and subsequently by GlcNAc T-II (MGAT2) to form bi-antennary complex type *N*-glycans [5]. The addition of β1-4 GlcNAc to the 4-linked central mannose residue of the trimannosyl core Man_3_GlcNAc_2_ of hybrid, bi-antennary or even tri-antennary *N*-glycans by GlcNAc T-III (GnT-III), encoded by the *MGAT3* gene, leads to the formation of bisecting GlcNAc *N*-glycan structures, thereby preventing further glycan branching [6]. Importantly, no other glycosyltransferase was shown to contribute to the formation of bisecting GlcNAc containing *N*-glycans as shown through genetic knockout studies in mice [7, 8]. In regards to ovarian cancer, the presence of this glycan motif was described in membrane and secreted glycoproteins from serous ovarian cancer specimens [9, 10] and *MGAT3* expression was shown to be elevated in ovarian cancer cell lines and tissue when compared to normal counterparts [11, 12].

Mechanisms underlying *MGAT3*/ bisecting GlcNAc expression in cancer have not been analyzed in detail, especially in ovarian cancer. Our previous findings have indicated that the relative ion intensities of bisecting GlcNAc-type bi-antennary *N*-glycans correlate with *MGAT3* expression in ovarian cancer cells [12]. Moreover, *MGAT3* expression can be increased upon 5-aza-2′-deoxycytidine (5-Aza) treatment, indicating an epigenetic regulation of *MGAT3* through DNA methylation [12]. This is in line with mouse experiments suggesting a transcriptional impact of DNA methylation at the CpG island of *MGAT3* during epithelial-to-mesenchymal transition [13].

Whilst aberrant glycosylation on membrane proteins and lipids has been well documented in several cancers [14, 15], the consequence of epigenetic regulation on specific glycosylation alteration remains largely unexplored. There are a few examples in the literature on DNA methylation-based transcriptional regulation of glycosyltransferase-encoding genes with almost all of them using DNA methyltransferase inhibitor 5-Aza [12, 16–20]. However, specific examples for 5-Aza-imposed DNA methylation leading to elevated glycosyltransferase-encoding gene expression and, more importantly, to the presence of the corresponding glycan motif on glycoproteins are rather limited. One example is the epigenetic regulation of alpha 2,3 sialyltransferase 6 (*ST3Gal6*) and its glycosidic product Lewis x which was reconstituted by 5-Aza in the colon cancer cell line HCT15 [16] and confirmed in the serous ovarian cancer cell line OVCAR3 with no additional analysis performed on CpG island DNA methylation [19].

In this study, we investigated whether DNA methylation of *MGAT3* gene in ovarian cancer can lead to the presence of corresponding *N*-glycosidic products bearing bisecting GlcNAc and how *MGAT3* expression impacts on cancer patient survival.

## RESULTS

### Human *MGAT3* contains two large CpG islands and is regulated by DNA methylation at the transcription starting site (TSS) in ovarian cancer cells

**Figure 1:**
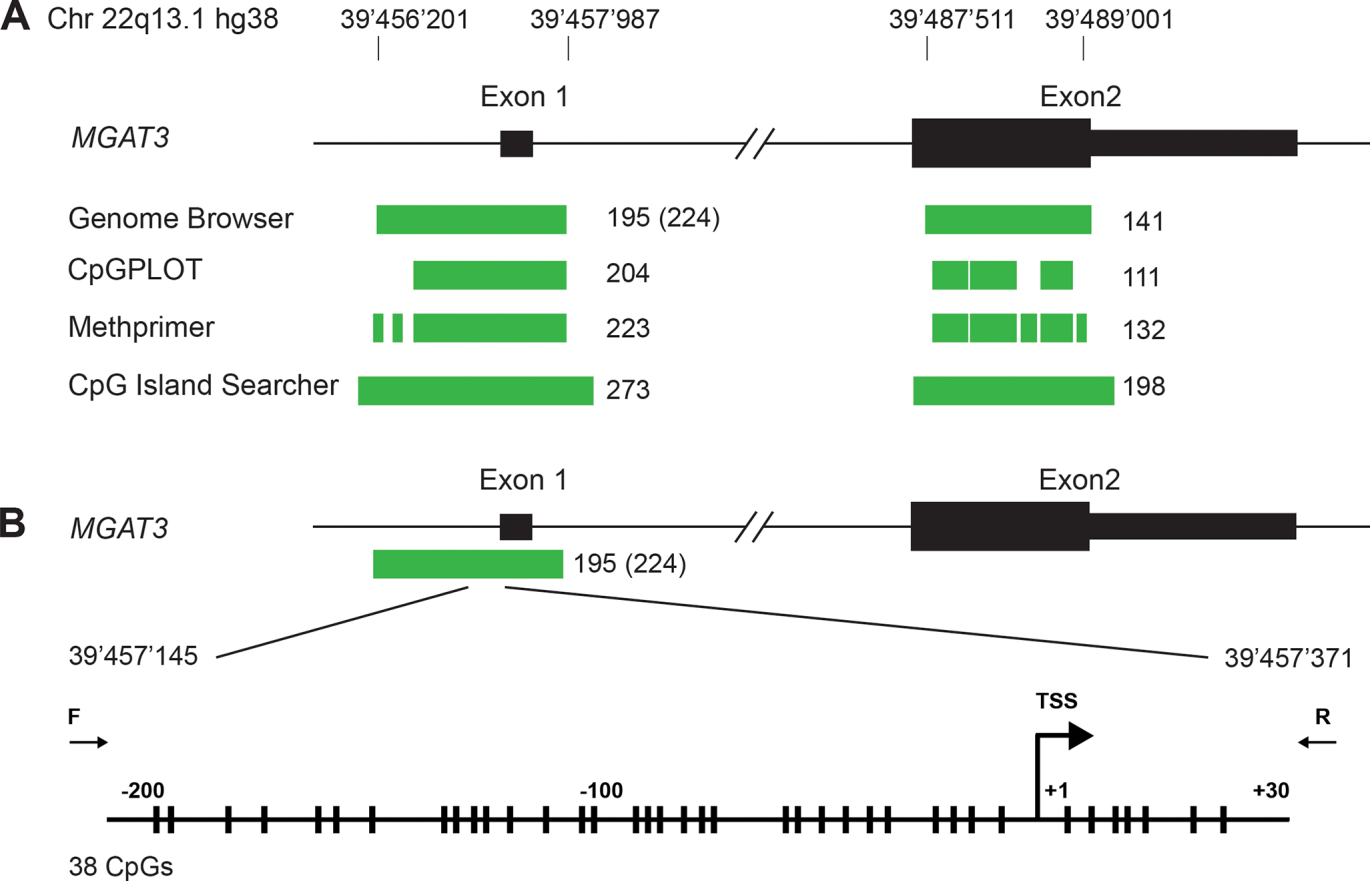
Human *MGAT3* gene contains two CpG Islands (**A**) CpG island analysis of the genomic locus of *MGAT3*. Exon 1 and 2 are shown in black (open reading frame depicted as larger bar), predicted CpG islands are depicted in green. (**B**) Genomic region analyzed in detail around the TSS is magnified. Individual CpGs are depicted with vertical bars and the TSS is indicated with a kinked arrow. Positions of forward and reverse primer used for bisulfite sequencing are indicated.

Our previous study suggested a correlation between bisecting GlcNAc containing *N*-glycoproteins and *MGAT3* expression, which might be linked to the methylation status of the *MGAT3* promoter in ovarian cancer cell lines [12]. However, the detailed analysis of the human *MGAT3* promoter in terms of the presence of a CpG island and the DNA methylation thereof was not addressed. Moreover, the reconstitution of bisecting GlcNAc in cells with negligible *MGAT3* expression upon treatment with 5-Aza was not shown. Thus, we sought to analyze the *MGAT3* genomic region for the presence of CpG islands in more detail. We used four different web-based bioinformatic engines to search for a CpG island in the *MGAT3* gene (UCSC Genome Browser, CpGPLOT, Methprimer and CpG island searcher [21–24]). All four programs predicted two CpG islands at the transcription start site (TSS) of the *MGAT3* gene. One CpG island encompasses exon 1 and the second is located within Exon 2 of *MGAT3* (Figure 1A). The length and CpG content vary depending on the program used for prediction but is within the range of 220 CpGs in 1600 bp for CpG island 1 and 120 CpGs in 1500 bp for CpG island 2 (Figure 1A, Supplementary Table 1). Since most changes in DNA methylation occur in a region of −250/+250 bp relative to the TSS [25], we analyzed the DNA methylation status around the TSS of *MGAT3* (Figure 1B). The high density of CpGs in the CpG island 1 rendered it difficult to design primers devoid of CpG dinucleotides. We found one pair that amplified 230 bp (−200/+30 relative to the TSS) of which both primers did not contain CpGs (Figure 1B). The amplified region contained 38 CpGs, 31 upstream and 7 downstream of the *MGAT3* TSS.

**Figure 2:**
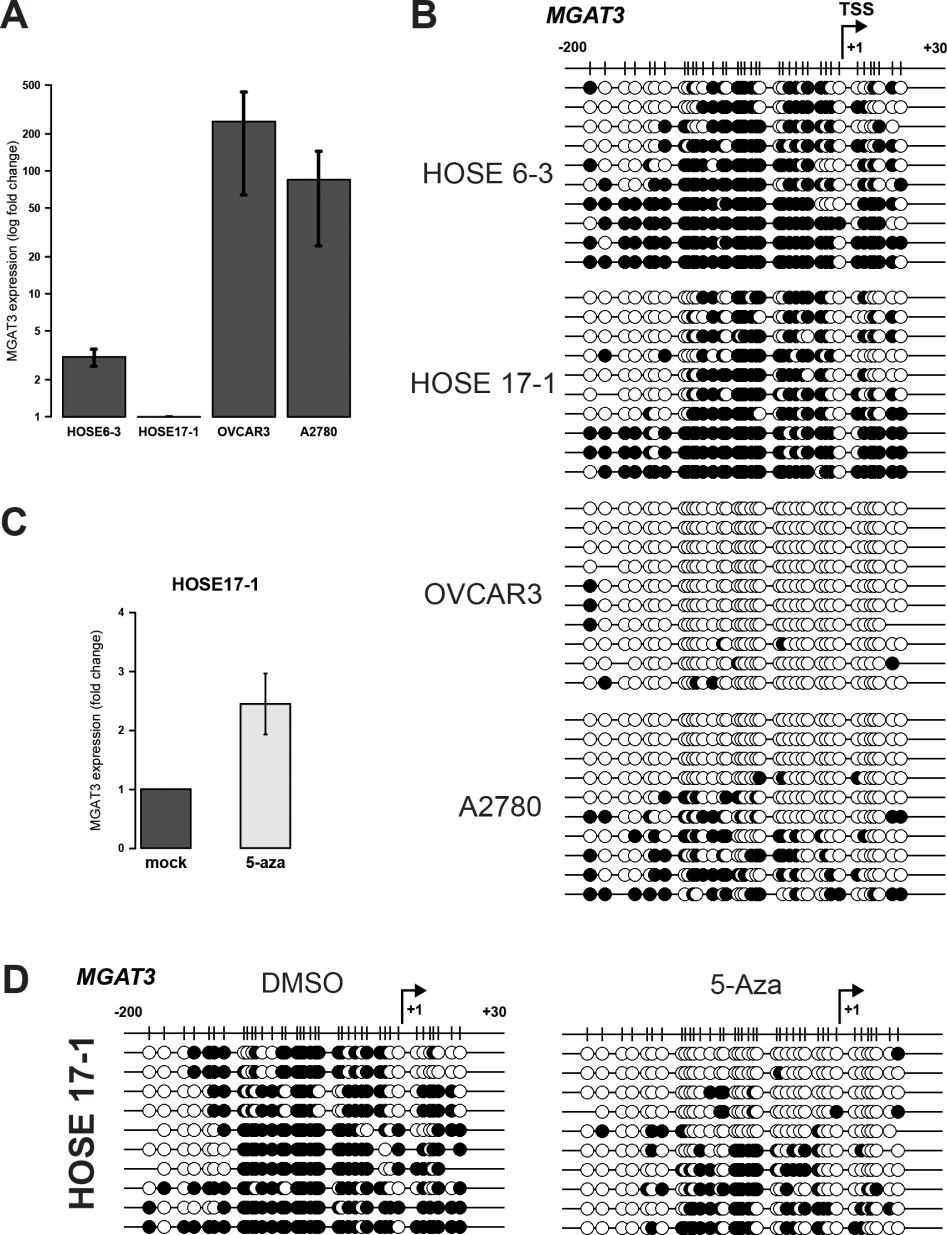
DNA methylation at −200/+30 from the TSS corroborates with *MGAT3* expression in HOSE and ovarian cancer cells (**A**) Relative *MGAT3* gene expression analyzed by RT-qPCR. *MGAT3* expression was normalized to logarithmic mean of three independent reference genes (*HSPCB*, *SDHA*, and *YWHAZ*). (**B**) Bisulfite sequencing result illustrated with black (methylated CpG) and white (unmethylated CpG) circles. (**C**) *MGAT3* expression is induced by the inhibition of DNA methyltransferases (+ 5-Aza). Gene expression was analyzed by RT-qPCR 72 hours after the addition of 5-Aza. (**D**) Bisulfite sequencing result of the *MGAT3* genomic region from HOSE17-1 cells analyzed in (**C**). *MGAT3* expression data are provided as mean and standard deviation of three independent experiments.

The analysis of *MGAT3* promoter DNA methylation was performed in normal human ovarian surface epithelial cell lines (HOSE6-3 and HOSE17-1) and two serous ovarian cancer cell lines (OVCAR3 and A2780), showing low and high *MGAT3* expression, respectively (Figure 2A). Bisulfite sequencing revealed hypermethylation of the analyzed region in HOSE6-3 and HOSE17-1 (68.1 ± 6.3% and 58.8 ± 6.8%, respectively) cells compared to hypomethylation in OVCAR3 and A2780 cells (2.7 ± 1.0% and 21.9 ± 6.1%, respectively) (Figure 2B). This demonstrates that DNA methylation around the TSS of *MGAT3* correlates with gene expression.

We have previously shown that *MGAT3* expression can be induced by inhibition of DNA methyltransferases using 5-Aza in HOSE cells [12] (Figure 2C). Importantly, here we demonstrate that overall DNA methylation around the TSS was reduced upon treatment with 5-Aza in HOSE17-1 cells (mock 67.8 ± 3.9% *versus* 5-Aza 26.6 ± 6.1%, *p* = 0.0162), indicating a direct relationship between TSS methylation and *MGAT3* expression (Figure 2D).

### DNA methylation at the TSS regulates expression of *MGAT3* and bisecting GlcNAc in ovarian cancer cell lines

**Figure 3:**
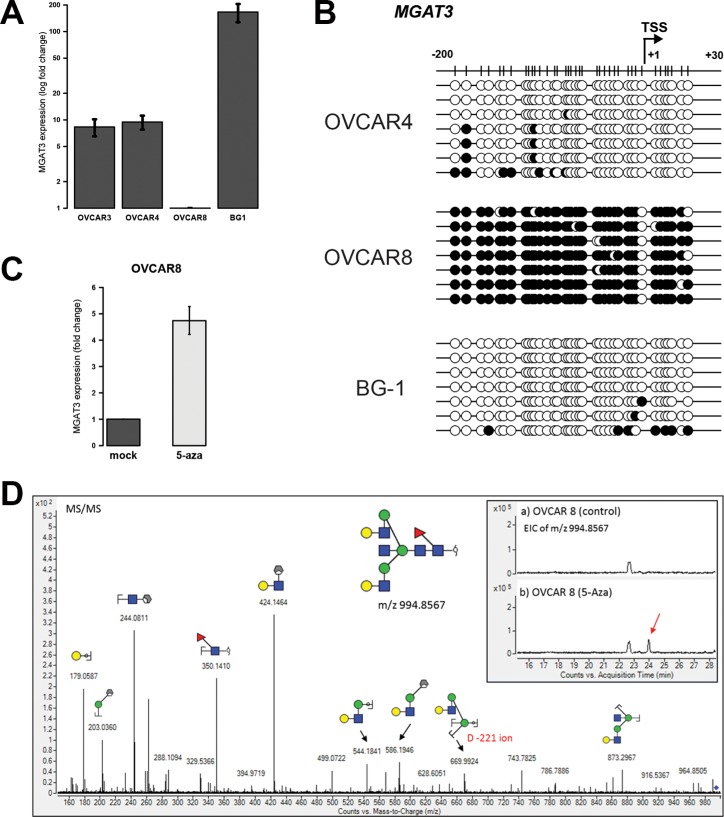
DNA methylation at −200/+30 from the TSS correlates with *MGAT3* expression in other ovarian cancer cells and bisecting GlcNAc is induced by DNA methyltransferase inhibition (**A**) *MGAT3* gene expression analysis by RT-qPCR. (**B**) Bisulfite sequencing result of corresponding cell lines profiled in A. Black circle (methylated CpG), white circle (unmethylated CpG). (**C**) *MGAT3* gene expression in OVCAR8 cells was analyzed by RT-qPCR after 72 hours of treatment with 5-Aza. (**D**) Reconstitution of bisecting GlcNAc upon 5-Aza treatments in OVCAR8 cells. *N*-Glycans were PNGase F-released from OVCAR8 membrane proteins used in (**C**) and analyzed by ESI-qTOF mass spectrometry. *MGAT3* expression data are provided as mean and standard deviation of three independent experiments.

Next, we addressed the question whether the regulation of *MGAT3* expression by DNA methylation of the TSS is a common feature of ovarian cancer cells. We analyzed *MGAT*3 expression and the corresponding DNA methylation in three additional serous ovarian cancer cell lines. High *MGAT3* expression was observed in OVCAR4 and BG-1 cells, whereas OVCAR8 cells showed unexpectedly low levels of *MGAT3* expression (Figure 3A).

In line with *MGAT3* expression data, TSS DNA hypomethylation was observed in cell lines with high *MGAT3* expression, BG-1 (3.8 ± 3.2%) and OVCAR4 (5.3 ± 2.4%) whereas DNA hypermethylation corroborate with decreased *MGAT3* expression in OVCAR8 (93.2 ± 1.0%) cells (Figure 3B). Inhibition of DNA methyltransferases by 5-Aza treatment resulted in an increased expression of *MGAT3* in OVCAR8 cells (Figure 3C). Importantly, mass spectrometric analysis of *N*-glycans released from membrane glycoproteins revealed the presence of a bisecting GlcNAc-containing glycan structure with m/z 994.8567, which was induced in OVCAR8 cells by the addition of 5-Aza (Figure 3D, insert). The presence of the bisecting GlcNAc was further verified using MS/MS fragmentation whereby the D-221 fragment ion at m/z 669.99, previously reported to be diagnostic for bisecting biantennary GlcNAc-type *N*-glycans [12], was found to be present in the MS/MS spectra (Figure 3D). Bisecting GlcNAc-containing glycan structures which were shown to be present on other ovarian cancer cells [26] were not detected (Supplementary Figure 2).

**Figure 4:**
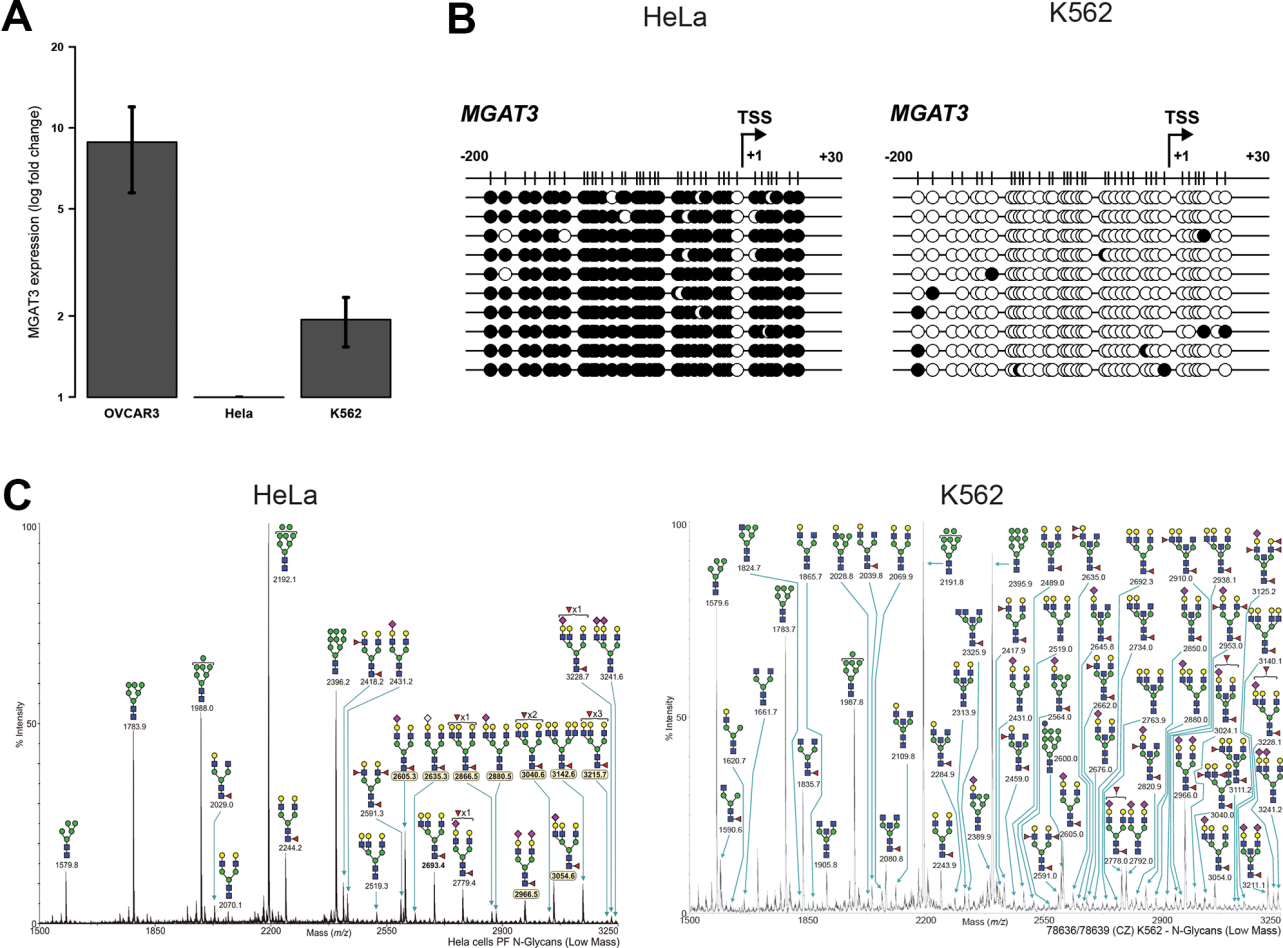
DNA methylation at −200/+30 from the TSS correlates with *MGAT3* and bisecting GlcNAc expression in non-ovarian cancer cell lines (**A**) Gene expression analysis by RT-qPCR. (**B**) Bisulfite sequencing showing black circle (methylated CpG) and white circle (unmethylated CpG). (**C**) The annotated *N*-glycomes of HeLa and K562 cells were accessed and downloaded through the Consortium for Functional Glycomics (CFG, www.functionalglycomics.org). Low molecular weight *N*-glycans are depicted.

We next expanded our analysis to investigate whether this strong association of DNA methylation, *MGAT3* gene and bisecting GlcNAc expression can be translated also to non-ovarian cancer cell lines of which *N*-glycan profiles are present. Therefore, we analyzed *MGAT3* expression in the cervical cancer cell line, HeLa and the leukemia cell line, K562, showing differential *MGAT3* expression (Figure 4A). In full concordance with gene expression, the TSS of *MGAT3* was highly methylated in HeLa (93.7 ± 0.9%) and hypomethylated in K562 (3.2 ± 0.8%) cells (Figure 4B). Interestingly, this pattern fully corroborates with the absence (HeLa) or presence (K562) of bisecting GlcNAc containing *N*-glycans as shown by glycoprofiling data obtained through the Consortium for Functional Glycomics (CFG, www.functionalglycomics.org) (Figure 4C), hence indicating a direct link between DNA methylation and bisecting GlcNAc expression, as shown in these two non-ovarian cancer cell lines.

### Ovarian cancer long-term survivors show lowest *MGAT3* expression

**Figure 5:**
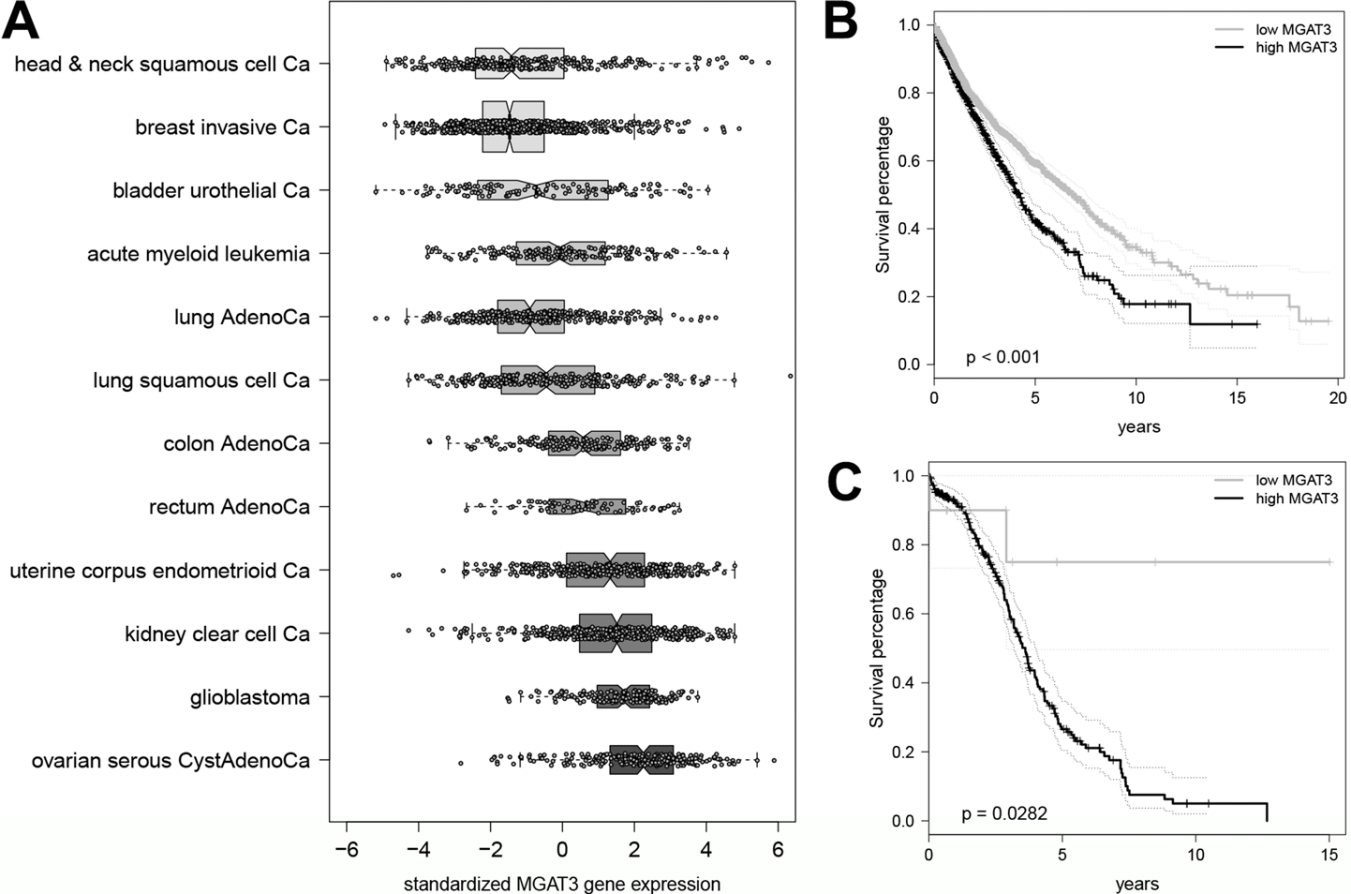
Highest expression of *MGAT3* in HGSOC among twelve TCGA datasets (**A**) Boxplot of *MGAT3* expression in TCGA PANCAN12 data set (*n* = 3587) sorted by median gene expression from lowest (head and neck squamous cell cancer) to highest (ovarian serious cystadenocarcinoma). Boxes for each data set are drawn with widths proportional to the square roots of the number of observations in the groups. (**B**) Elevated *MGAT3* expression is associated with poor survival in all cancer types. Kaplan-Meier curve for dichotomized *MGAT3* expression in TCGA PANCAN12 data set (*n* = 3477). (**C**) Kaplan-Meier curve for dichotomized *MGAT3* expression in HGSOC patients obtained from the TCGA PANCAN12 data set (*n* = 264).

Based on the strong relationship observed between DNA methylation, *MGAT3* gene and bisecting GlcNAc expression in various normal and cancer cell lines, we sought to reproduce our results in human cancer tissue samples. We analyzed the overall *MGAT3* expression among all cancers ≤ (*n* = 12) in the TCGA PANCAN12 dataset to address the question whether *MGAT3* expression is cancer-type specific (Figure 5). The *MGAT3* expression varied among all TCGA datasets from being the lowest in head & neck squamous cell carcinoma to being the highest in HGSOC. Moreover, the TCGA HGSOC cohort showed significantly elevated *MGAT3* expression compared to all remaining cancer types except for glioblastoma (*p* < 0.001, Figure 5A).

Since ovarian cancer is the gynecological cancer with the poorest prognosis and displayed highest *MGAT3* expression, we investigated whether *MGAT3* expression correlates with survival in all TCGA PANCAN12 cancer samples and in particular, HGSOC (Figure 5B and 5C). The tree-based model selected a threshold (*MGAT3* ≤ 1.67) in regards to survival separating all cancers (*n* = 3477) in low and high *MGAT3* expression (*p* < 0.001) (Figure 5B). Interestingly, the investigation of the HGSOC cohort (*n* = 264) revealed a small number of patients (*n* = 10) which showed lowest *MGAT3* expression (*MGAT3* ≤ −1.177) and displayed a flattened out survival curve, indicating long-term survivorship of up to 15 years (*p* = 0.0282, Figure 5C). Multivariate analysis identified *MGAT3* expression as an independent prognostic marker (*p* = 0.047, HR = 1.118, 95% CI 1.000–1.248) for overall survival. Residual disease and age at diagnosis but not FIGO stage, grade, lymphatic as well as venous invasion were identified as independent prognostic markers.

### Basal-like breast cancers also display high *MGAT3* expression with inverse correlation to DNA methylation

**Figure 6:**
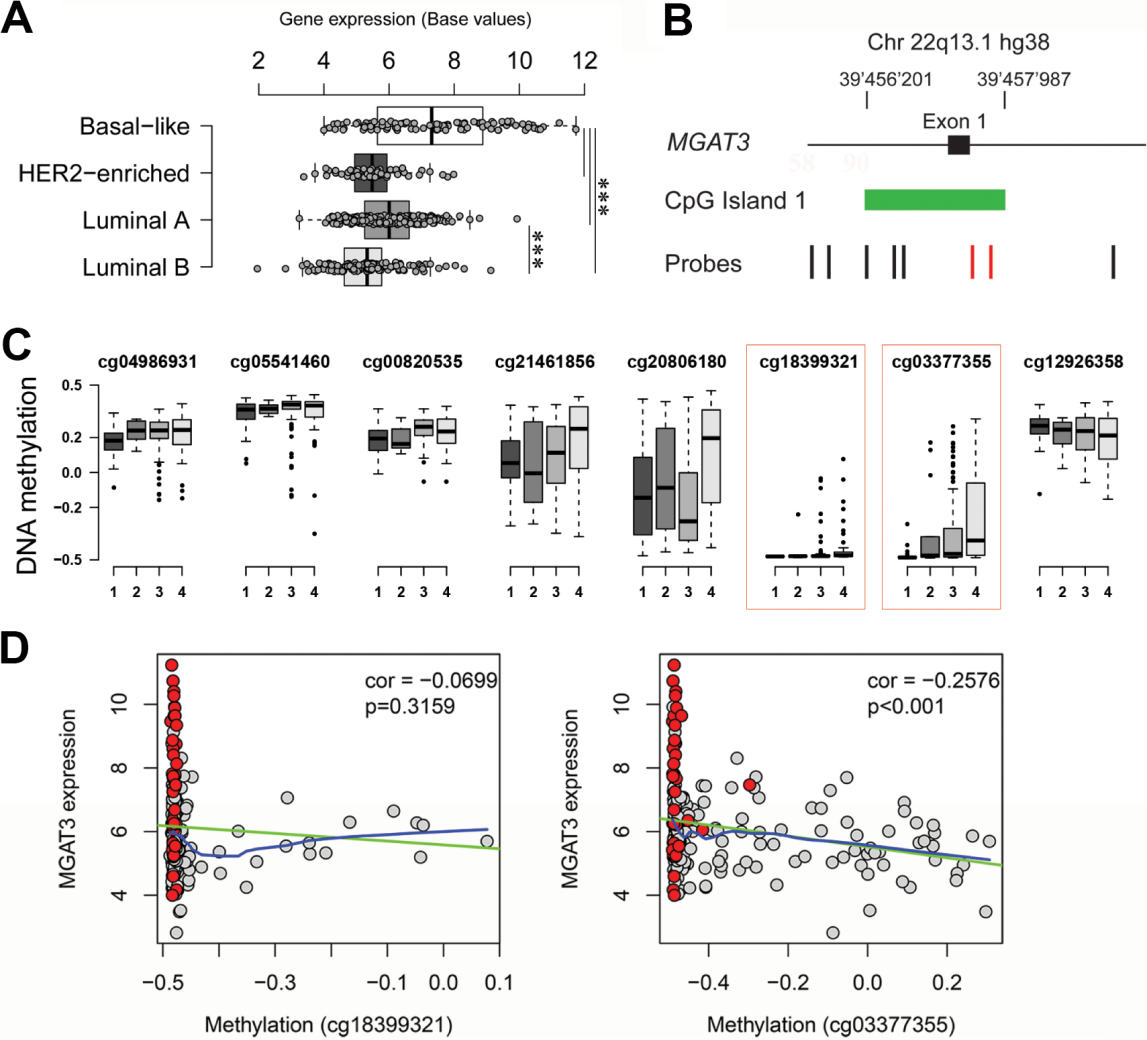
Elevated *MGAT3* expression correlates with DNA hypomethylation in TCGA breast cancer data (**A**) Boxplot showing significantly elevated *MGAT3* expression in basal-like breast cancer compared to HER2-enriched, luminal A and luminal B breast cancer (***p-value < 0.001). (**B**) Genomic localization of probes of the 450K HumanMethylation Bead chip (black and red vertical bars) at exon 1 (black box) of *MGAT3*. CpG island 1 is shown in green. (**C**) Boxplot displaying degree of DNA methylation (normalized by centering to 0) in the TCGA breast cancer cohort for selected probes (same order left to right as annotated in A). Degree of DNA methylation is shown for basal-like (1), HER2-enriched (2), luminal-A (3), and luminal-B (4) breast cancer (abscissa). Probes significantly discriminating four breast cancer groups are highlighted in the red dotted box. (**D**) Correlation plot for TCGA breast cancer samples matching *MGAT3* expression and DNA methylation of probes cg18399321 and cg03377355 (red highlighted in A and B). Basal-like are highlighted in red while remaining breast cancer samples are grey. Correlation and *p*-value are shown in each plot. Linear and locally weighted polynomial regressions are shown in green and blue, respectively.

We next analyzed the correlation between DNA methylation and *MGAT3* expression in human tumors in more detail. The 450K DNA methylation data (Infinium HumanMethylation450 BeadChip, Illumina, San Diego, United State of America) is not yet available for HGSOC and the 27K human DNA methylation data provided only one probe (cg18399321) for HGSOC, which is completely de-methylated (Supplementary Figure 1). Therefore we unfortunately had to exclude HGSOC from this analysis. Since recent genomic and transcriptomic data of basal-like breast cancers suggest a close molecular relation to HGSOC [27], we further analyzed *MGAT3* expression in the TCGA breast cancer dataset (*n* = 514) and compared basal-like (*n* = 98), HER2-enriched (*n* = 58), luminal A (*n* = 231) and B (*n* = 127) types of breast cancers. Interestingly, we observed significantly elevated *MGAT3* expression (ANOVA *p* < 0.001) in basal-like cancers compared to all other breast cancer subtypes and similar *MGAT3* expression levels as in HGSOC (Figure 6A, Supplementary Table 2).

The breast cancer dataset was also analyzed by HumanMethylation450 BeadChip data in regards to DNA methylation and *MGAT3* expression in matched tissue samples. The location of the CpG probes in the 450K DNA methylation dataset was dispersed over CpG island 1 of *MGAT3* (Figure 6B). We then analyzed the methylation scores of the indicated individual probes in breast cancer separated by the four subtypes. The red boxed probes (cg18399321 and cg03377355), which are in close proximity to the genomic region analyzed by bisulfite sequencing in cell lines above, displayed changes in methylation score when we compared basal-like (Lane 1) with HER2 enriched (lane 2), Luminal A (lane 3) and Luminal B (lane 4) (Figure 6C). A significant reduction of DNA methylation was observed in basal-like samples when compared to the remaining three breast cancer subtypes for probes cg18399321 (ANOVA *p* = 0.0189) and cg03377355 (ANOVA *p* < 0.001). Surrounding probes presented equally high degrees of DNA methylation among all four breast cancer subtypes (Figure 6C, Supplementary Table 2).

We then addressed the correlation between probe methylation score and *MGAT3* expression in four breast cancer types. Basal-like samples were almost completely hypomethylated. A negative correlation was found for both probes with only cg03377355 being statistically significant (*p* < 0.001), indicating that the genomic region at this site of *MGAT3* may also be involved in epigenetic regulation of *MGAT3* expression (Figure 6D, Supplementary Table 2).

### *MGAT3* expression inversely correlates with DNA methylation in human cancer samples

**Figure 7:**
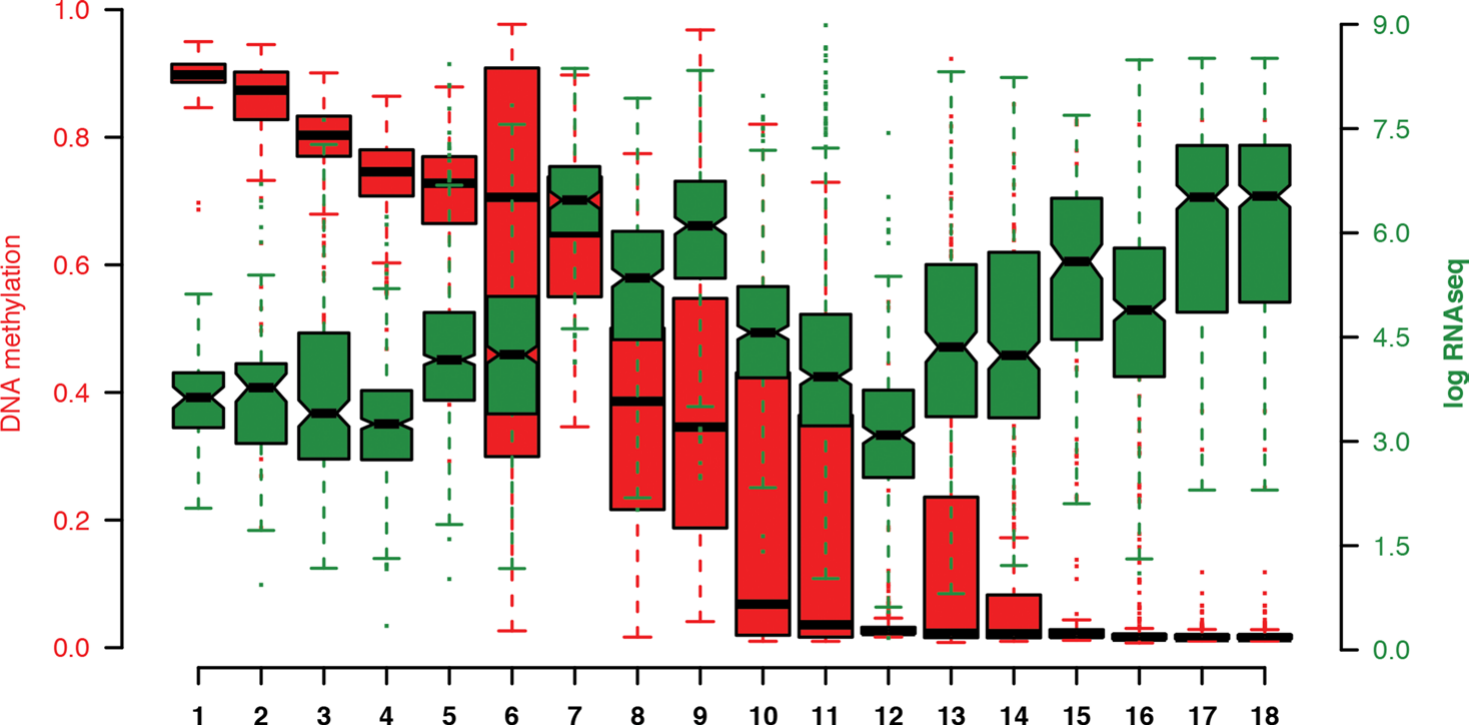
Boxplots of methylation values sorted by median (red) and *MGAT3* expression (green) obtained by 450K DNA methylation array and RNA sequencing through the cBioPortal for cancer genomics, respectively Each boxplot represents values obtained from the TCGA database of matched DNA methylation (red) and RNAseq data (green). DNA methylation data represent as the beta-value of the probe that is most negatively correlated with the expression of the *MGAT3* gene. Black horizontal lines represent the median with the box representing the 25th and 85th percentiles, the whiskers the 5th and 95th percentiles, outliers shown by dots in corresponding color (red, green). 1) Kidney chromophobe, 2) Adrenocortical carcinoma, 3) Kidney renal papillary carcinoma, 4) Prostate carcinoma, 5) Breast invasive carcinoma, 6) Skin cutaneous melanoma, 7) Pancreatic adenocarcinoma, 8) Colorectal adenocarcinoma, 9) Kidney renal clear cell carcinoma, 10) Lung adenocarcinoma, 11) Liver hepatocellular carcinoma, 12) Cervical squamous cell carcinoma and endocervical adenocarcinoma, 14) Bladder urothelial carcinoma, 15) Uterine corpus endometrial carcinoma, 16) Lung squamous cell carcinoma, 17) Papillary Thyroid carcinoma, 18) Thyroid carcinoma.

Since we observed a correlation between *MGAT3* expression and DNA methylation in breast cancer datasets, we investigated whether this finding can be translated to other human cancers (Figure 7). Genomic data from 18 TCGA epithelial cancer datasets were analyzed where both DNA methylation and matched *MGAT3* expression data were available (*n* = 6118 samples). The kidney chromophobe cohort had the lowest sample size (*n* = 66), while the invasive breast carcinoma displayed the cohort with the largest number of samples (*n* = 735) in this study. In concordance with our results on cell lines as well as TCGA breast cancer dataset analysis, samples with DNA hypermethylation had generally lower levels of *MGAT3* expression in the TCGA datasets. In contrast, DNA hypomethylated samples revealed a trend towards elevated *MGAT3* expression (Figure 7), thus resulting in an inverse correlation for *MGAT3* expression (multiple R^2^ 0.2945; *p* = 0.01997).

## DISCUSSION

Alterations in protein glycosylation occur frequently in cancer and can regulate tumor development and progression. Aberrant glycans might be explained by changes of glycosyltransferase gene expression, chaperone function, mislocalization of glycosyltransferases, un-/availability of sugar donors and altered glycosidase activity [28, 29]. Our current data supports the relationship of epigenetic regulation on *MGAT3* expression through DNA methylation and the presence of bisecting GlcNAc on *N*-glycans in HGSOC. We expanded our study to epithelial cancer types other than HGSOC, demonstrating that DNA methylation and *MGAT3* expression corroborates among almost all epithelial cancer types and has influence on disease outcome, independent of the cancer subtype. This relationship can be reproduced in basal-like breast cancer, a histotype resembling the molecular signature of HGSOC.

To our knowledge, this is the first detailed analysis of human *MGAT3* gene which illustrates two CpG islands located in both exons consisting of roughly 200 and 100 individual CpGs, sharing similarities with the organization of the mouse *MGAT3*, despite being smaller in size than the human counterpart [13]. In contrast, human *MGAT5* does not have a CpG island [19], whereas mouse *MGAT5* has one CpG island at exon 1 [13], indicating that *MGAT3* might have an evolutionary conserved type of epigenetic regulatory mechanism.

We have previously shown that the product of GnT-III (encoded by the *MGAT3* gene), bisecting GlcNAc, is present on ovarian cancer cells but is absent on human ovarian surface epithelial cells [12]. We thus hypothesized that the differential gene and glycan expression might be regulated by DNA methylation. Since epigenetic changes primarily occur in close proximity to the TSS [25], we specifically analyzed DNA methylation around the TSS of *MGAT3*. Elevated DNA methylation correlated with decreased *MGAT3* expression in human ovarian surface epithelial and *vice versa* in ovarian cancer cells. Moreover, the *MGAT3* expression in hypermethylated cells was reconstituted upon treatment with 5-Aza, correlating with loss of DNA methylation at the TSS. We also confirmed our findings using a broader panel of ovarian and non-ovarian cancer cells. Importantly, addition of 5-Aza not only restored *MGAT3* expression in OVCAR8 cells, but also resulted in the expression of bisecting GlcNAc containing *N*-glycans, thereby clearly indicating a direct relationship of DNA methylation-dependent bisecting GlcNAc expression. Interestingly, we did not detect a diverse range of bisecting GlcNAc-containing *N*-glycan structures in OVCAR8, apart from the non-sialylated bi-antennary bisecting GlcNAc *N*-glycan, which was also reported to be the most prominent form analyzed previously in other ovarian cancer cell lines such as OVCAR3 [26]. This demonstrates that the regulation of *MGAT3* seems to be unique amongst glyco-genes involved in the *N*-glycosylation pathway, since other genes (*MGAT4A* and *MGAT5*) were shown to be indirectly regulated by DNA methylation [30]. A similar regulatory mechanism was only observed in the case of *B4GALNT2*, an enzyme involved in the synthesis of blood group carbohydrate antigens [17]. *B4GALNT2* promoter DNA hypermethylation in colorectal cancer correlated with decreased gene expression and the absence of the glycan Sd^a^ on the cell surface. Similar findings were also observed for *ST3GAL6* [16]. However, the promoter of *B4GALNT2* and *ST3GAL6* seems to be hypermethylated in cancer in contrast to the *MGAT3* promoter, which is hypomethylated.

Recent genomic and transcriptomic data suggest that basal-like breast and HGSOC are intriguingly similar in terms of *TP53*, *BRCA1/2* and *RB1* mutation status and also based on transcriptional correlations [27]. In addition to that, clinicopathological characteristics are in full concordance with this molecular phenotype and both cancer types are highly aggressive [31]. Interestingly, an inverse correlation between DNA methylation of *MGAT3* and gene expression was also observed in basal-like breast cancer. Since this data indicates a tissue-specific regulation of *MGAT3*/bisecting GlcNAc expression in human cancers, the role of *MGAT3*/bisecting GlcNAc in cancer development or metastasis is very likely to differ within different types of human cancers. Although bisecting GlcNAc was shown to be involved in E-cadherin- [32], epidermal growth factor (EGF)- [33], Wnt/ beta-catenin- [34] and integrin/focal adhesion kinase (FAK)- [35] mediated signaling pathways. However, underlying molecular mechanisms altered by MGAT3/bisecting GlcNAc in HGSOC are yet to be identified, for instance with the help of CRISPR/ Cas9-mediated deletion of *MGAT3* in HGSOC cells.

Overall, our data demonstrate, for the first time, a direct relationship between DNA methylation around the TSS of *MGAT3*, *MGAT3* gene expression and the presence of bisecting GlcNAc in HGSOC and basal-like breast cancer. It is evident that epigenetic regulation through DNA methylation may primarily be associated with *MGAT3*/ bisecting GlcNAc expression, however, other mechanisms such as nucleosome positioning [36] and histone modifications [37] are also possible.

The prognosis of HGSOC has not changed over the past decades despite advances in aggressive cytoreduction, targeted therapy and increasing knowledge about underlying genetic mutations. In fact, it still remains as the gynecological cancer with the worst prognosis, reflected by a five-year survival of only 20%. However, there are instances where HGSOC patients are present with a long-term survivorship of over ten years. To date, only few analyses, including our own, have examined this patient cohort despite its vast importance. Our analysis in regards to *MGAT3* expression of patients from the large TCGA dataset indicates that the loss of *MGAT3* results in a dramatic plateau in the survival curves of HGSOC. This observation has, so far, not been associated with any biomarker or treatment measure in ovarian cancer. Therefore, our data strongly supports further investigation into the role of *MGAT3* as a modulator of long-term survival, reflecting a yet unknown underlying biology.

## MATERIALS AND METHODS

### CpG island analysis

Sequence information of human *MGAT3* (Hg38, Chr22: 39448663-39498392) was extracted from the UCSC genome browser and subjected to Genome Browser, CpGPLOT, Methprimer and CpG island searcher [21–24] in order to predict CpG islands in this region using default settings.

### Cell culture and treatments

HOSE6-3, HOSE17-1, OVCAR3, A2780, OVCAR4, OVCAR8, BG-1, HeLa and K562 cells were cultured in RPMI supplemented with 10% fetal bovine serum and penicillin/streptomycin. Cells were treated with 5-aza-2′-deoxycytidine (5-Aza) as described previously [38]. All cell line-based experiments were performed independently on three different passages. Cells were tested routinely for mycoplasma infection.

### Reverse-transcription quantitative polymerase chain reaction (RT-qPCR)

Total RNA was extracted from exponentially growing cells using the ReliaPrep™ RNA Cell Miniprep system according to the manufacturers' instructions (Promega, Dübendorf, Switzerland). Total RNA (1μg) was reverse-transcribed using iScript cDNA synthesis kit (Bio-Rad Laboratories AG, Cressier, Switzerland). RT-qPCR was performed on *MGAT3* and reference genes *HSPCB*, *SDHA*, and *YWHAZ* in 10 μl reactions containing 10 ng cDNA (initial total RNA), 400nM forward and reverse primer, nuclease free water and 1× SensiFast^™^ with low ROX as the reference dye (Bioline, Biolabo scientific instruments, Châtel-St-Denis, Switzerland) on a ViiA^™^ 7 Real-Time PCR System (Applied Biosystems, Thermo Fisher Scientific, Reinach, Switzerland). Quantitative PCR was performed in triplicates and analyzed as recently described [26, 39].

### Bisulfite sequencing

Genomic DNA was extracted from exponentially growing cells as described [38]. An amount of 1 μg genomic DNA was bisulfite converted using the EZ Methylation-Gold Kit (Zymo Research Inc., Lucerna-Chem AG, Luzern, Switzerland). Bisulfite converted DNA was subjected to PCR reaction using MyTaq™ HS DNA polymerase (Bioline, Biolabo scientific instruments, Châtel-St-Denis, Switzerland). Primers devoid of CG dinucleotides for bisulfite sequencing were designed using bisulfite primer seeker (http://www.zymoresearch.com/tools/bisulfite-primer-seeker). PCR reactions contained: 1× MyTaq reaction buffer, MyTaq HS polymerase, 300 nM forward primer (5′-gggggaggggagggggtt-3′), 300 nM reverse primer (5′-cctccccccacccccacttc-3′) and nuclease free water to a final volume of 10 μl. PCR was performed by initial denaturation at 95°C for 1 min, followed by 40 cycles of 95°C for 15 sec, 66°C for 15 sec, 72°C for 20 sec and a final amplicon elongation at 72°C for 10 min. The PCR product (230 bp) was visualized on a 2% agarose gel and purified from the gel using the Wizard SV Gel and PCR clean up system (Promega, Dübendorf, Switzerland). The purified PCR product was cloned into pGEM^®^-T Easy (Promega) and plasmid minipreps (PureYield™ Plasmid MiniPrep System, Promega) from individually grown colonies were sequenced using either T7 or SP6 primer (Sanger DNA sequencing service, Source Bioscience, Berlin, Germany). Bisulfite sequencing data were analyzed and visualized using the QUMA web-based software tool [40]. Results are provided as mean ± standard error of methylated CpGs between DNA sequences. Student *t*-test was applied for statistical analysis.

### Membrane protein extraction and release of *N*-glycans from OVCAR8 cells

Cell membranes of OVCAR8 cells were prepared as previously described [12]. Briefly, approximately 1 × 10^6^ OVCAR 8 and 5-Aza-treated OVCAR8 cells were washed with PBS, pelleted through centrifugation and re-suspended with lysis buffer (50 mM Tris-HCl, 100 mM NaCl, 1 mM EDTA and protease inhibitor) at pH 7.4 prior to homogenization. Cellular debris and unlysed cells were removed by centrifugation and the supernatant was subjected to ultracentrifugation at 120,000 g to pellet the cell membranes. Cell membranes were re-suspended in Tris binding buffer containing Triton ×-114 for phase partitioning of membrane proteins. The detergent layer containing the membrane proteins were acetone-precipitated and solubilized in 8 M urea. *N*-glycans were prepared and purified as previously described [12]. Briefly, membrane proteins and glycoprotein standard (fetuin) were spotted onto a polyvinylidene diflouride (PVDF) membrane, air-dried overnight at room temperature and stained. Protein spots were placed in a 96-well microtiter plate and treated with PNGase F enzyme to release the *N*-glycans. Released glycans were collected and treated with 100 mM ammonium acetate (pH 5.0) to ensure a complete regeneration of the reducing terminus. The released *N*-glycans were reduced to alditols with 2M NaBH_4_ in 50 mM KOH and the reduction was quenched using glacial acetic acid. *N*-glycan alditols were purified using cation exchange chromatography and subsequent washings with methanol were performed for the removal of residual borate. The purified *N*-glycan alditols were re-suspended in 15 μl of MilliQ water prior to mass spectrometry analysis.

### LC-ESI-qTOF-MS/MS of released *N*-glycan alditols

The analysis of released *N*-glycan's was performed using an Agilent UHPLC system, consisting of a binary pump, autosampler, thermostat and column oven (Agilent Series 1290, Agilent Technologies, Waldbronn, Germany) connected to a high-resolution hybrid mass spectrometer (MS) (Agilent Series 6540 Q-TOF, Agilent Technologies, Santa Clara, CA, USA). The front end of the MS was equipped with a jet stream electrospray ionization source (ESI) (Agilent Technologies, Santa Clara, CA, USA). The chromatographic separation of *N*-glycans was modified from Anugraham *et al.*, 2014 and applied to a conventional HPLC method. In brief, 15 μL of samples were injected onto a Hypercarb porous graphitized carbon capillary column (3 μm particle size; 2.1 × 150 mm, Thermo Fisher Scientific, Runcorn, UK). The mobile phase was made up of 10 mM ammonium bicarbonate (A) and 9:1 acetonitrile/water with 10 mM ammonium bicarbonate (B). The column was re-equilibrated with starting mobile phase condition for 8 minutes and the column compartment was kept at 40°C. The separation of *N*-glycans was carried out over a linear gradient method (0–5 min; 1 % B, 40 min; 25 % B; 42 min; 50 % B and 42.5 min; 80 % B, hold for 1.5 min) with a column flow of 0.4 mL/min. The ESI source was operated in negative mode with the following parameter settings: nebulizer pressure-35 psig, nozzle voltage- 0 V, sheath gas flow- 11 L/min, sheath gas temperature −375°C, drying gas flow- 8 L/min, drying gas temperature- 250°C, capillary voltage and fragmentor voltage at 4000 V and 175 V, respectively. To obtain the highest mass accuracy, a constant flow of internal reference masses (m/z 119.0363 and 981.9956) was applied on the end plate of the ESI Source. MS scan (m/z 100-1700 at 1.5 Hz) and MS/MS (m/z 100-1700 at 3 Hz) spectra were acquired with triggered data-dependent algorithm with static exclusion range of m/z 100-600 in 2 GHz mode. Precursor ions were isolated with a width of m/z 1.3 in Q1 and fragmented with a variable collision energy based on slop x (m/z) /100 + offset (slop = 3, offset = 2). The precursor ion threshold was set to 10 000 counts with a maximum of 15 precursor ions per cycle (cycle time 5.8 sec).

The MS data were analyzed with MassHunter B.06.00 (Agilent) and the monosaccharide compositions of monoisotopic masses were determined using GlycoMod tool (http://web.expasy.org/glycomod/) with a mass error of ± 20 ppm [41]. The proposed bisecting GlcNAc *N*-glycan structural feature was assigned and interpreted based on diagnostic MS/MS fragmentation patterns previously reported for negative ion mode MS [12] and annotated using GlycoWorkBench 2.1 software [42]. In addition, blank samples and fetuin samples which served as quality control for sample preparation were randomly measured within each sequence run and checked for background contamination.

### TCGA data analysis

TCGA data sets were accessed through the UCSC Cancer Genomics Browser website [43, 44] (https://genome-cancer.ucsc.edu/proj/site/hgHeatmap/). Gene expression and matched DNA methylation (Figure 7) indicating the RNA sequencing data and Beta-value [45] respectively, were accessed using the cBioPortal for Cancer Genomics [46] using cgdsr, a R-based application programming interface (API) that provides a basic set of R functions for querying the Cancer Genomics Data Server (CDGS) hosted by the Memorial-Sloan-Kettering Cancer Center (MSKCC).

*MGAT3* gene expression was analyzed using TCGA_PANCAN12_exp_HiSeqV2 dataset. *MGAT3* gene expression and methylation were analyzed using the breast cancer dataset TCGA_BRCA_exp_HiSeqV2 and TCGA_BRCA_hMethyl450 (Infinium HumanMethylation 450K).

Overall survival dependent on *MGAT3* expression was investigated using Kaplan-Meier curves. *MGAT3* expression was dichotomized using tree based model partitioning (“ctree” in R package “party”). Computations of the predicted survivor curves were used for Cox proportional hazards regression model (R package “survival”). Multivariate analysis was performed with fitted Cox proportional hazards regression model (R package “survival”) including *MGAT3* expression, FIGO stage, grade, residual disease, lymphatic invasion, venous invasion, and age at initial diagnosis. *MGAT3* expression was adjusted for these covariates. Results were presented as hazard ratios (HR) with 95% confidence interval (CI) and corresponding *p*-values. In order to explore associations between DNA methylation and *MGAT3* expression, Pearson correlation was calculated.

Comparisons between cancer subgroups were performed using one-way ANOVA. A *p* value < 0.05 was considered significant. All statistical evaluations were done using the statistical software R version 3.1.3 (www.R-project.org).

## SUPPLEMENTARY MATERIALS FIGURES AND TABLES


