# Supplementary material for: Epigenetic activation of MGAT3 and corresponding bisecting GlcNAc shortens the survival of cancer patients
Source: Oncotarget. 2016 Jul 12;7(32):51674–86. doi: 10.18632/oncotarget.10543 (PMC5239506; doi:10.18632/oncotarget.10543)
Supplement: Supplementary file 1 [file oncotarget-07-51674-s001.pdf]

Epigenetic activation of *MGAT3* and corresponding bisecting GlcNAc shortens the survival of cancer patients

Supplementary Materials

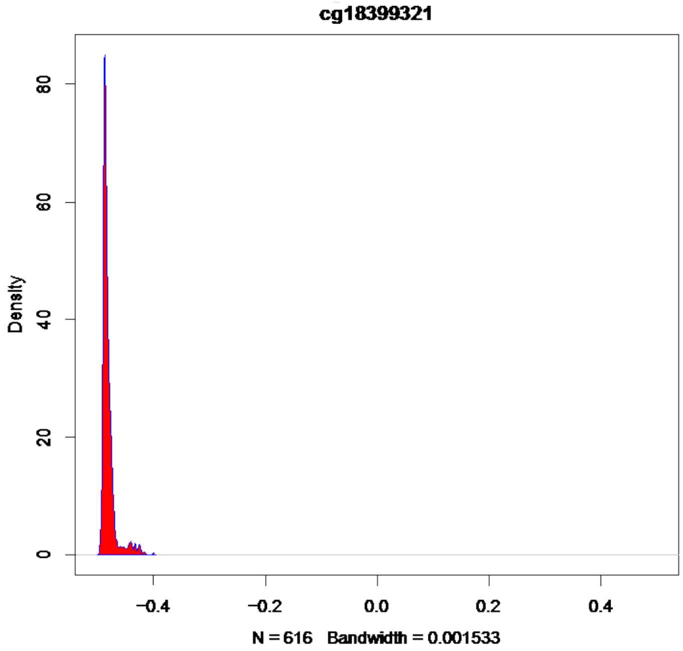

Supplementary Figure S1: Histogram displaying degree of methylation of TCGA serous ovarian cystadenocarcinoma at probe cg18399321 of the 27K Illumina human Methylation Array.

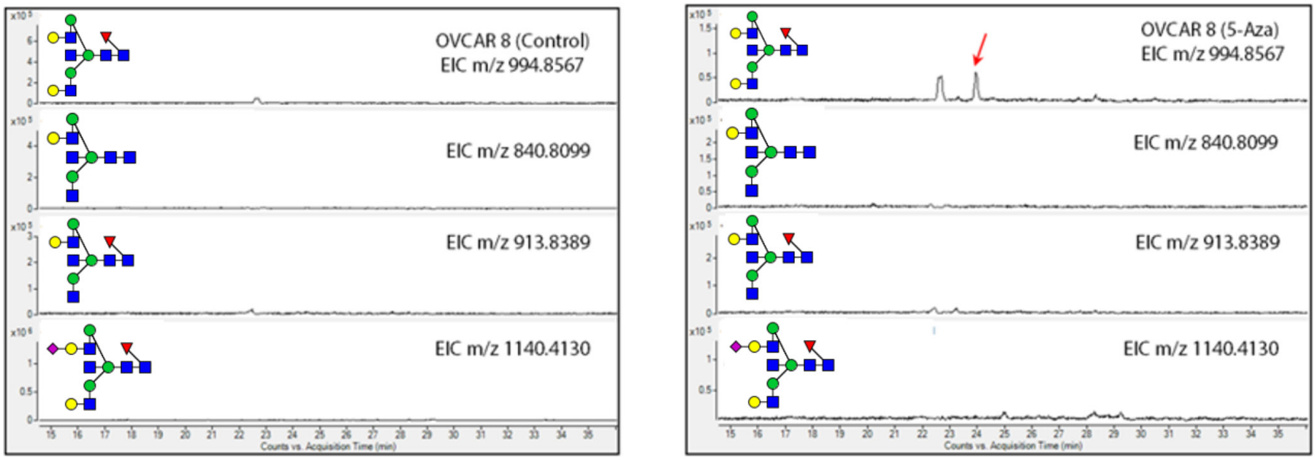

Supplementary Figure S2: Extracted Ion Chromatogram (EIC) of untreated OVCAR8 cells (left) and 5-Aza treated OVCAR8 cells (right).

Supplementary Table S1: Precise genomic location on chromosome 22q13 hg38 of *MGAT*3 CpG Islands are listed

	CpG Island #	Start	End	Length	CpGs
**Genome Browser**	1	39456201	39457987	1787	195(224)
	2	39487511	39489005	1495	141
**CPG PLOT**	1	39456673	39458072	1400	204
	2	39487545	39487887	344	35
	3	39487911	39488415	505	55
	4	39488564	39488808	245	21
**Methprimer**	1	39456165	39456318	154	11
	2	39456527	39456646	119	8
	3	39456673	39458072	1400	204
	4	39487545	39487887	344	35
	5	39487911	39488415	505	55
	6	39488438	39488543	107	7
	7	39488564	39488808	245	21
	8	39488816	39488984	169	14
**CpG Island Searcher**	1	39456418	39458412	1995	273
	2	39487384	39489238	1855	198

Default settings for CpG island query were GC content > = 50%, length > = 200 bp and ration of observed to expected CGs > = 0.6 in the case of Genome Browser, Cpgplot and Methprimer. CpG island searcher used GC content > = 55% and ratio observed to expected GCs > = 0.65.

Supplementary Table S2: Correlating *MGAT3* expression and DNA methylation at two probes in close proximity to the transcription start site of *MGAT3*

MGAT3 expression PAM50_mRNA_nature2012 (*n* = 514); **Figure 6A**
ANOVA *p*-value < 0.0001

Basal-like (*n* = 98) | **HER2-enriched (*n* = 58)** | **Luminal A (*n* = 231)** | **Luminal B (*n* = 127)**

Pairwise comparison	Basal-like	HER2-enriched	Luminal A
**HER2-enriched**	< 0.0001		
**Luminal A**	< 0.0001	0.051	
**Luminal B**	< 0.0001	1.0	< 0.0001

DNA methylation (*n* = 208); Figure 6C and 6D

Basal-like (*n* = 40) | **HER2-enriched (*n* = 14)** | **Luminal A (*n* = 108)** | **Luminal B (*n* = 46)**

probe cg18399321 (ANOVA *p*-value 0.0189)			
Pairwise comparison	**Basal-like**	**HER2-enriched**	**Luminal A**
**HER2-enriched**	1		
**Luminal A**	1	1	
**Luminal B**	0.013	0.880	0.119
probe cg03377355 (ANOVA *p*-value < 0.0001)			
Pairwise comparison	**Basal-like**	**HER2-enriched**	**Luminal A**
**HER2-enriched**	0.0593		
**Luminal A**	0.0593	0.9113	
**Luminal B**	9.5E-06	0.1973	0.0201
